# First report of mixed *Trypanosoma cruzi* discrete typing units infection in *Triatoma phyllosoma* in the peri-urban environment of Oaxaca, Mexico

**DOI:** 10.1590/0037-8682-0449-2023

**Published:** 2024-03-25

**Authors:** Dulce Concepción Domínguez-Cruz, Doireyner Daniel Velázquez-Ramírez, Zendy Evelyn Olivo-Vidal, José Antonio De Fuentes-Vicente, Héctor Ochoa-Díaz-López

**Affiliations:** 1 El Colegio de la Frontera Sur, Departamento de Salud, San Cristóbal de Las Casas, CHIS, México.; 2 El Colegio de la Frontera Sur, Departamento de Salud, Villahermosa, TAB, México.; 3 Universidad de Ciencias y Artes de Chiapas, Instituto de Ciencias Biológicas, Tuxtla Gutiérrez, CHIS, México.

**Keywords:** American trypanosomiasis, Triatomines, Discrete typing unit, Southeastern Mexico, Istmo de Tehuantepec

## Abstract

**Background:**

Chagas disease, a zoonosis transmitted mainly by hematophagous insects of the subfamily Triatominae, is caused by *Trypanosoma cruzi*, classified into six discrete typing units (DTUs: TcI-TcVI and Tcbat).

**Methods:**

Insect vectors were collected from 84 human dwellings in the municipality of Santo Domingo Tehuantepec, Oaxaca, Mexico; 4.76% were infested. DTUs were determined using conventional and nested PCR.

**Results:**

The infection rate was 43.6%. All insects were infected with TcI while one specimen showed mixed infection with TcII.

**Conclusions:**

This is the first report of *T. cruzi* mixed infection in *Triatoma phyllosoma*, its main vector in the study region.

Chagas disease is a zoonotic disease caused by the parasite *Trypanosoma cruzi*, widely distributed throughout the Americas. According to the World Health Organization (WHO), it is listed as one of the most important vector-borne diseases (VBDs), which currently affects approximately six million people worldwide^1^. Owing to large genetic variation, *Trypanosoma cruzi* has been classified into six discrete typing units: TcI-TcVI and TcBat^2^. All DTUs are found throughout the Americas; TcI is the most prevalent in both wild and domestic cycles and strongly associated with cardiac involvement, while DTUs TcII, TcV, and TcVI are associated with cardiodigestive disorders^3^.

Mexico is one of the countries most affected by Chagas disease, with a significantly increased number of cases in recent years, most of which are concentrated in the southeast region of the country^4^, where environmental and social conditions contribute to vector-borne transmission. In particular, the state of Oaxaca has among the highest morbidity and mortality rates for Chagas disease. Currently, 157 triatomine species have been recognized and described worldwide (154 living species and three fossil species). Brazil has the greatest biodiversity of triatomines (more than 60 species)^5^ ^,^ ^6^, followed by Mexico (more than 30 species), of which 19 are epidemiologically relevant because their ability to invade and colonize human dwellings^7^. The following species have been documented in Oaxaca: *T. phyllosoma, T. pallidipennis, T. mazzottii, T. barberi,* and *T. dimidiata* ^8^ *.*

*Triatoma phyllosoma* is one of the main vectors of *T. cruzi* in the Istmo de Tehuantepec region where infection rates of 38-40% have been documented^8^ ^-^ ^10^. Although TcI has been reported, very few studies have focused on identifying the circulating DTUs in this region^10^. The objective of this study was to detect *T. cruzi* infection and to identify circulating DTUs in *T. phyllosoma* in the peri-urban domestic environment of the municipality of Santo Domingo Tehuantepec, Oaxaca, Mexico.

The study was conducted in the region of the Istmo of Tehuantepec in the Mexican state of Oaxaca, in the periphery (Lieza district) of the municipality of Santo Domingo Tehuantepec (16° 19' 41'' N, 95° 14' 42'' W) (Figure 1). This area is located 55 m above sea level. The vegetation is mainly low deciduous forest, the climate is warm sub-humid, with temperatures ranging from 20 to 30 °C, and the annual precipitation is between 700 and 1,000 mm. The corresponding local authorities were informed about the project objectives, and houses located on the outskirts of the Lieza district were selected. Triatomine sampling was conducted in March 2022 inside the dwellings (intra-domicile). In total, 84 households were visited, and triatomines were captured from four (4.76%). Collections were made by directly searching for cracked walls, beds, mattresses, furniture, concrete floors, and soil. Each specimen (adult and nymph) was individually placed in plastic jars lined with filter paper and labeled with the collection site information. The triatomines were then transferred to the “Laboratory of Molecular and Nutrigenomic Epidemiology, Emergent, Epidemic and Metabolic Diseases Group (EEEMA), Department of Health, El Colegio de la Frontera Sur (ECOSUR), San Cristobal,” where the sex and species were identified using the Lent and Wygodzinsky keys^5^.

**FIGURE 1:**
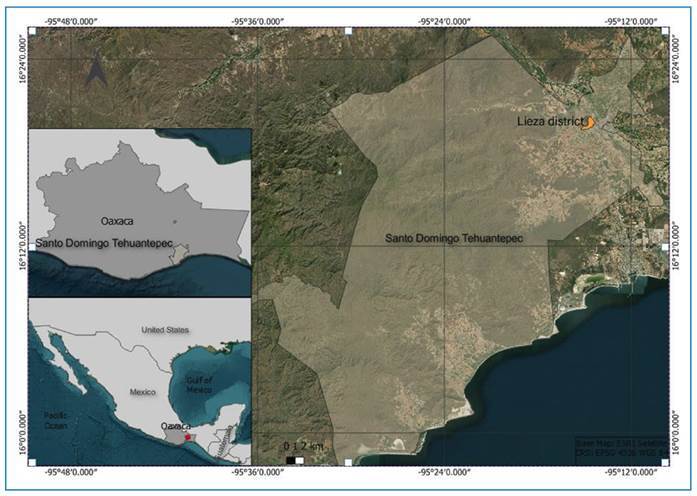
Location of Lieza district, Santo Domingo Tehuantepec, Oaxaca, Mexico.

The intestinal contents of each triatomine were extracted by abdominal incision and treated with DNA/RNA Shield™ (Zymo Research), according to the manufacturer’s instructions, vortexed vigorously, and stored at 4°C until processing. DNA was extracted using the Quick-DNA™ Miniprep Plus Kit (Zymo Research) according to the manufacturer’s instructions.

DNA amplification was performed using conventional polymerase chain reaction (cPCR) and nested PCR (nPCR) on three previously described and widely used gene regions of interest for identifying the *T. cruzi* DTUs (Table S1)^11^: the intergenic region of the miniexon gene (SL-IR), the D7 domain region of the 24Sα ribosomal RNA gene, and the A10 nuclear fragment. For cPCR, primers TCC, TCI, and TCII were used to amplify 350 bp (TcI) or 300 bp (TcII, TcV, and TcVI) fragments of the SL-IR region. For nPCR, primers D75 and D76 (first round) and D76 and D71 (second round) were used to amplify 125 bp (TcIII), 140 bp (TcII and TcVI), 140 /145 bp (TcIV), and 125 or 125/140 bp (TcV) fragments of the D7 domain region. For the nuclear fragment region, A10 was amplified by nPCR using primers Pr1 and P6 (first round) and Pr1 and Pr3 (second round), which amplified fragments of 580 bp (TcII) and 525 bp (TcVI) (Figure S1). Reactions were set up with 12 µl of Thermo Scientific PCR Master Mix 2x, 1 µl of each primer, 30 ng of DNA (1 µl of nuclease-free water was used as a negative control), and nuclease-free water was added to complete a final volume of 25 µl. Amplification was performed on a Bio-Gener ELVE32G thermal cycler, and the PCR products were visualized under UV light on 2.0% agarose gels stained with GelRed (Biotium).

In total, 45 triatomines were caught in the dwellings, four (8.9%) fifth instar nymphs and 41 (91.1%) adults, of which 31 (75.6%) were female and 10 (24.4%) were male. All specimens were identified as *T. phyllosoma*. The intestinal contents of 39 triatomines were processed for detecting *T. cruzi* infection, as six had insufficient material for DNA extraction. *Trypanosoma cruzi* infection was detected in 17/39 (43.6%) triatomines, including 13/17 (76.5%) females and 4/17 (23.5%) males; no infection was detected in the collected nymphs (Table S2). Molecular typing of *T. cruzi* revealed that 16/17 (94.2%) of the positive samples were TcI, while 1/17 (5.8%) had mixed TcI-TcII infection as indicated by amplification of the miniexon intergenic region (SL-IR) (Figure 2). The DTUs of the mixed-infection samples corresponded to TcI and TcII, as shown in Figure 3.

**FIGURE 2:**
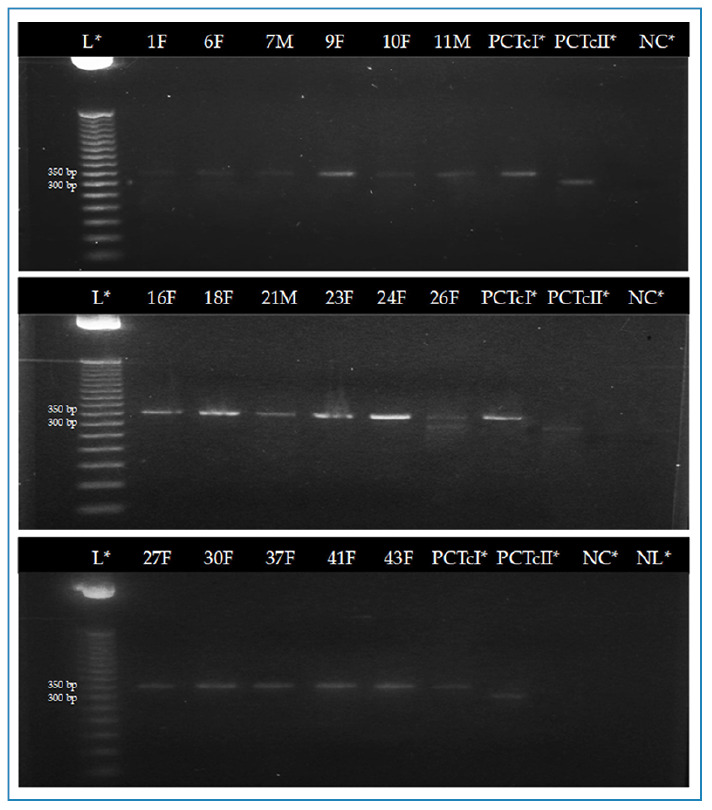
Visualization of conventional PCR amplification products for the miniexon gene from the intestinal contents of *Triatoma phyllosoma* collected from the Istmo of Tehuantepec, Oaxaca, Mexico, for identifying *Trypanosoma cruzi*. In 16 samples, 350 bp amplified fragments corresponding to TcI were observed, while in sample 26F, two fragments of 350 bp and 300 bp were observed, corresponding to a mixed infection with TcI-[TcII-TcVI (undetermined)]. L*: Ladder; PCTcI*: positive control TcI; PCTcII*: positive control TcII; **NC:** negative control; **NL:** not loaded.

**FIGURE 3:**
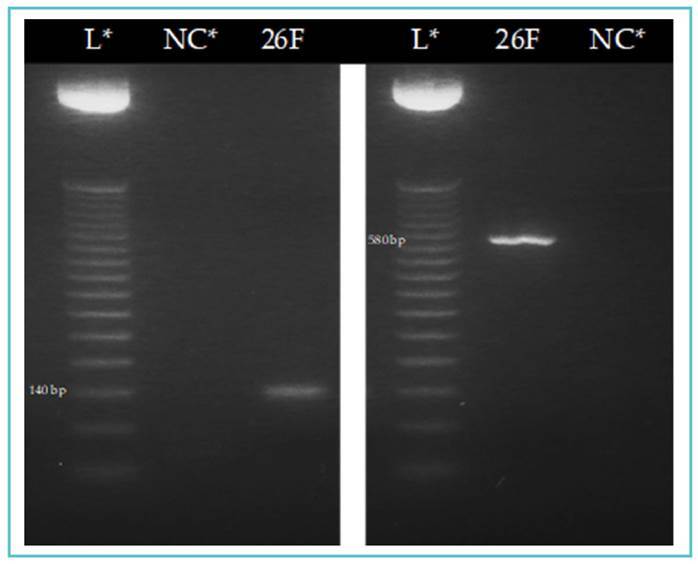
Visualization of the nested PCR amplification products of the D7 domain and the A10 nuclear region from the intestinal contents of sample 26F of *Triatoma phyllosoma* collected from the Istmo of Tehuantepec, Oaxaca, Mexico, for identifying *Trypanosoma cruzi*. **a)** Amplification of a 140 bp fragment of the 24Sα ribosomal RNA gene indicates the presence of TcII and TcVI DTUs. **b)** Amplification of a 580 bp fragment of the nuclear region confirms the presence of TcII. L*: ladder; NC*: negative control.

Currently, vector-borne *T. cruzi* is associated with habitat fragmentation and human activities such as deforestation and urbanization. These activities contribute to the dispersal of triatomines from a wild environment to a semi-urban or urban environment through attraction to light, search for food, and shelter. This behavior is more common in endemic regions and mainly affects households in urban peripheries and in precarious housing conditions^9^.

Historically, the State of Oaxaca has been a relevant setting for studying Chagas disease in Mexico. In 1940, Dr. Luis Mazzotti documented the first cases of the acute disease in the community of Tejumulco, Oaxaca^12^. From 2000-2016, high rates of chronic cases and disease-related mortality of 1.1-4.4 per 100,000 people were reported^4^. Although the reasons for this are still largely unknown, the great diversity of triatomine species and the lack of effective vector control programs are considered to play key roles.

In our study, adult triatomines and fifth instar nymphs belonging to *T. phyllosoma* were found. This is consistent with previous studies that identified *T. phyllosoma* as the dominant species in the southeastern region of Oaxaca^7^ ^,^ ^8^. Similarly, in a study carried out in the Istmo de Tehuantepec, *T. phyllosoma* was identified as the only species present in the area^9^. The rate of *T. cruzi* infection in our study was 43.6%, which was within the range (40-49%) reported in previous studies conducted in Oaxaca^9^ ^,^ ^13^; however, it is slightly higher than that in a previous report from the Istmo de Tehuantepec (39%)^9^ ^,^ ^10^.

Regarding the distribution of DTUs, our research results are limited to the urban context of the Istmo de Tehuantepec. We mainly detected TcI and reported a mixed infection (TcI-TcII). Concerning the distribution of TcI, our report is similar to that of studies conducted in the Istmo region of Oaxaca, specifically in the municipality of Salina Cruz, where TcI has been isolated from *T. phyllosoma* ^10^. Therefore, our results support the available literature on the distribution of TcI in this geographic region. Although *T. phyllosoma* is one of the most important vectors for transmitting *T. cruzi* in the Istmo de Tehuantepec region, there are no previous reports on *T. phyllosoma* infected by TcII. Our findings represent the first report of this DTU circulating in the domestic peri-urban cycle of this species. There are currently reports of mixed infections ( TcI and TcVI) in *T. dimidiata* captured in the Valles Centrales region of Oaxaca State^10^. Mixed *T. cruzi* infections have important epidemiological implications, including difficulty in characterizing the variants present in the infected individual, disease progression and severity, as well as resistance to the drugs of choice^2^ ^,^ ^14^. To the best of our knowledge, some orders of wild mammals (Chiroptera, rodents, and primates) have been infected with DTU II; however, how this genotype reaches humans remains largely unknown. Further research is thus necessary to determine the potential sources of transmission in the study area^15^.

Although our study provides information on the distribution of *T. cruzi* DTUs in urban environments, understanding the parasite-vector dynamics and their impact on pathogenesis in humans and other domestic animals is necessary. Currently, there are reports of associations between DTUs and the clinical manifestations of Chagas disease^3^. However, *T. cruzi* genetic diversity has not been considered in epidemiological surveillance. The inclusion of *T. cruzi* typing would thus facilitate a better understanding of the molecular epidemiology of this parasite in Mexico and other geographic regions where Chagas disease is endemic.
