# Supplementary material for: First report of mixed Trypanosoma cruzi discrete typing units infection in Triatoma phyllosoma in the peri-urban environment of Oaxaca, Mexico
Source: Rev Soc Bras Med Trop. 2024 Mar 25;57:e00703-2024. doi: 10.1590/0037-8682-0449-2023 (PMC10962353; doi:10.1590/0037-8682-0449-2023)
Supplement: Supplementary file 1 [file 1678-9849-rsbmt-57-e00703-2024-supp1.pdf]

Table S1: PCR conditions and concentration of the primers used.

Region	Primers	Concentration	PCR conditions*			
			D	A	E	C
**SL-IR**	TCC (CCCCCCTCCCAGGCCACACTG)	1µM	94ºC/5´	-	-	-
	TCI (GTGTCCGCCACCTCCTTCGGGCC)	1µM	94ºC/1´	61ºC/1´	72ºC/1´	35
	TCII (CCTGCAGGCACACGTGTGTGTG)	1µM	-	-	72ºC/7´	-
**Domain D7**	D75 (GCAGATCTTGGTTGGCGTAG)	5µM	94ºC/5´	-	-	-
	D76 (GGTTCTCTGTTGCCCCTTTT)	5µM	94ºC/1´	64ºC/1´	72ºC/1´	2
			94ºC/1´	62ºC/1´	72ºC/1´	2
			94ºC/1´	60ºC/1´	72ºC/1´	2
			94ºC/1´	58ºC/1´	72ºC/1´	35
			-	-	72ºC/5´	-
	D71 (AAGGTGCGTCGACAGTGTGG)	5µM	94ºC/5´	-	-	-
	D76 (GGTTCTCTGTTGCCCCTTTT)	5µM	94ºC/1´	60ºC/1´	72ºC/1´	3
			94ºC/1´	57ºC/1´	72ºC/1´	3
			94ºC/1´	55ºC/1´	72ºC/1´	35
			-	-	72ºC/5´	-
**Nuclear fragment A10**	Pr1 (CCGCTAAGCAGTTCTGTCCATA)	5µM	94ºC/5´	-	-	-
	P6 (GTGATCGCAGGAAACGTGA)	5µM	94ºC/1´	60ºC/1´	72ºC/1´	35
			-	-	72ºC/7´	-
	Pr1 (CCGCTAAGCAGTTCTGTCCATA)	5µM	94ºC/5´	-	-	-
	Pr3 (CGTGGCATGGGGTAATAAAGCA)	5µM	94ºC/1´	60ºC/1´	72ºC/1´	35
			-	-	72ºC/7´	-

*D (denaturation), A (alignment), E (extension) and C (number of cycles). Based on previous studies[11].
