# Supplementary figures and images for: First report of mixed Trypanosoma cruzi discrete typing units infection in Triatoma phyllosoma in the peri-urban environment of Oaxaca, Mexico
Source: Rev Soc Bras Med Trop. 2024 Mar 25;57:e00703-2024. doi: 10.1590/0037-8682-0449-2023 (PMC10962353; doi:10.1590/0037-8682-0449-2023)

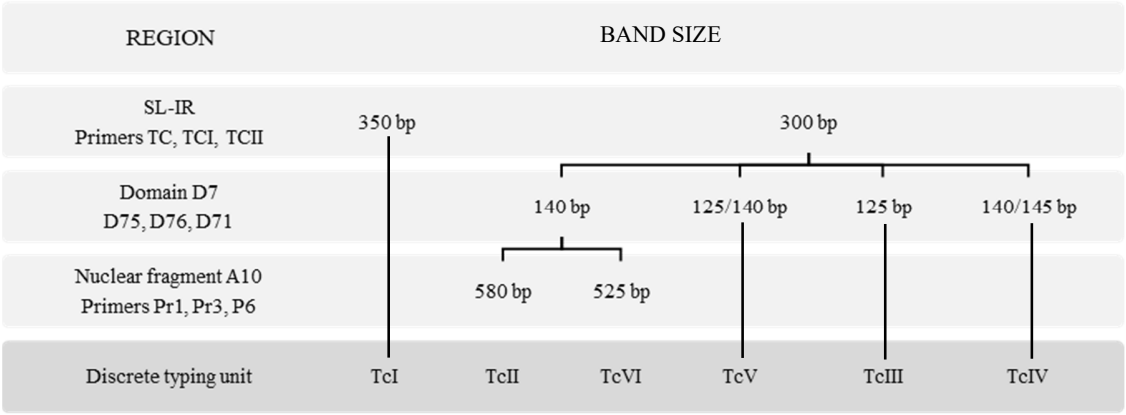

**Figure S1:** Diagram of the primers used.

Supplement: Supplementary file 2 [file 1678-9849-rsbmt-57-e00703-2024-supp2.pdf]
