# Supplementary material for: First report of mixed Trypanosoma cruzi discrete typing units infection in Triatoma phyllosoma in the peri-urban environment of Oaxaca, Mexico
Source: Rev Soc Bras Med Trop. 2024 Mar 25;57:e00703-2024. doi: 10.1590/0037-8682-0449-2023 (PMC10962353; doi:10.1590/0037-8682-0449-2023)
Supplement: Supplementary file 3 [file 1678-9849-rsbmt-57-e00703-2024-supp3.pdf]

Table S2: Characterization and infection of Triatomines (*Triatoma phyllosoma*) collected in urban domestic environment from Lieza district in Santo Domingo de Tehuantepec, Oaxaca.

Number of triatomines	Stage of development	*T. cruzi* infection
**1F**	Adult Female	Positive, TcI
**2M**	Adult Male	Negative
**3M**	Adult Male	Negative
**4N**	Stage 5 nymph	Negative
**5F**	Adult Female	Excluded Sample
**6F**	Adult Female	Positive, TcI
**7M**	Adult Male	Positive, TcI
**8N**	Stage 5 nymph	Negative
**9F**	Adult Female	Positive, TcI
**10F**	Adult Female	Positive, TcI
**11M**	Adult Male	Positive, TcI
**12N**	Stage 5 nymph	Negative
**13F**	Adult Female	Negative
**14M**	Adult Male	Negative
**15N**	Stage 5 nymph	Excluded Sample
**16F**	Adult Female	Positive, TcI
**17M**	Adult Male	Negative
**18F**	Adult Female	Positive, TcI
**19M**	Adult Male	Negative
**20F**	Adult Female	Negative
**21M**	Adult Male	Positive, TcI
**22F**	Adult Female	Negative
**23F**	Adult Female	Positive, TcI
**24M**	Adult Male	Positive, TcI
**25M**	Adult Male	Negative
**26F**	Adult Female	Positive, TcI-TcII
**27F**	Adult Female	Positive, TcI
**28F**	Adult Female	Negative
**29F**	Adult Female	Negative
**30F**	Adult Female	Positive, TcI
**31F**	Adult Female	Excluded Sample
**32F**	Adult Female	Excluded Sample
**33F**	Adult Female	Negative
**34F**	Adult Female	Excluded Sample
**35F**	Adult Female	Negative
**36F**	Adult Female	Negative
**37F**	Adult Female	Positive, TcI
**38F**	Adult Female	Negative
**39F**	Adult Female	Negative
**40F**	Adult Female	Excluded Sample
**41F**	Adult Female	Positive, TcI
**42F**	Adult Female	Negative
**43F**	Adult Female	Positive, TcI
**44F**	Adult Female	Negative
**45F**	Adult Female	Negative
